# Source Community and Assembly Processes Affect the Efficiency of Microbial Microcystin Degradation on Drinking Water Filtration Membranes

**DOI:** 10.3389/fmicb.2019.00843

**Published:** 2019-04-18

**Authors:** Marisa O. D. Silva, Peter Desmond, Nicolas Derlon, Eberhard Morgenroth, Jakob Pernthaler

**Affiliations:** ^1^Limnological Station, Department of Plant and Microbial Biology, University of Zurich, Zurich, Switzerland; ^2^Eawag, Swiss Federal Institute of Aquatic Science and Technology, Dübendorf, Switzerland; ^3^Institute of Environmental Engineering, ETH Zurich, Institute of Environmental Engineering, Zurich, Switzerland

**Keywords:** biofilm, microcystin, drinking water, membrane filtration, microbial communities, network analysis, operational taxonomic unit

## Abstract

Microbial biofilms in gravity-driven membrane (GDM) filtration systems can efficiently degrade the cyanotoxin microcystin (MC), but it is unclear if this function depends on the presence of MC-producing cyanobacteria in the source water habitat. We assessed the removal of MC from added *Microcystis aeruginosa* biomass in GDMs fed with water from a lake with regular blooms of toxic cyanobacteria (ExpL) or from a stream without such background (ExpS). While initial MC removal was exclusively due to abiotic processes, significantly higher biological MC removal was observed in ExpL. By contrast, there was no difference in MC degradation capacity between lake and stream bacteria in separately conducted liquid enrichments on pure MC. Co-occurrence network analysis revealed a pronounced modularity of the biofilm communities, with a clear hierarchic distinction according to feed water origin and treatment type. Genotypes in the network modules associated with ExpS had significantly more links to each other, indicating that these biofilms had assembled from a more coherent source community. In turn, signals for stochastic community assembly were stronger in ExpL biofilms. We propose that the less “tightly knit” ExpL biofilm assemblages allowed for the better establishment of facultatively MC degrading bacteria, and thus for higher overall functional efficiency.

## Introduction

Harmful cyanobacterial blooms (cyanoHABs) are increasingly threatening water quality in freshwater and coastal marine ecosystems (Huisman et al., 2018; Paerl, 2018). Some cyanobacterial strains, e.g., from the freshwater genera *Microcystis* and *Planktothrix* (Harke et al., 2016; Kurmayer et al., 2016), produce toxic intracellular secondary metabolites, such as microcystins (MCs). These compounds are a particular concern both in drinking and recreational water systems due to their acute and chronic toxicity (Azevedo et al., 2002; Greer et al., 2018). This has led WHO to establish a guideline value of 1 μg L^-1^ of the ubiquitous MC variant MC-LR in drinking water (WHO, 1998).

A number of studies have reported biological MC degradation in the water column (Zhang et al., 2017; Krishnan et al., 2018; Thees et al., 2018; Yang et al., 2018), in biofilms (Babica et al., 2005; Kohler et al., 2014), and in sediments (Holst et al., 2003; Chen X. et al., 2010) of freshwater habitats, and MC degrading bacterial strains from various phylogenetic lineages have been isolated (Rapala et al., 2005; Amé et al., 2006; Manage et al., 2009; Chen J. et al., 2010). One aerobic pathway of MC-LR degradation mediated by the *mlr* gene cluster has been identified in *Sphingomonas* sp. (Bourne et al., 1996). This gene encodes for three enzymes (MlrA, MlrB, and MlrC) that are responsible for the degradation process: first the cyclic peptide is linearized by cleavage of the Adda-Arg bond, and subsequently the MlrB and MlrC enzymes hydrolyze the linear peptide into smaller ones (Bourne et al., 2001; Dziga et al., 2013). Additional degradation pathways have been suggested based on the discovery of novel intermediates and degradation semi-products (Amé et al., 2006; Edwards et al., 2008; Hashimoto et al., 2009; Zhang et al., 2010; Dziga et al., 2017). Moreover, other, hypothetical mechanisms for MC degradation have also been proposed, e.g., alkaline proteases or glutathione S-transferases (Takenaka and Watanabe, 1997; Mou et al., 2013), and the observation of MC degradation under anoxic conditions (Holst et al., 2003; Chen and Chen, 2010; Chen X. et al., 2010) strongly speaks for alternative metabolic strategies. Currently, it is not clear if and to which extent the known MC degrading strains are responsible for this process in complex microbial assemblages.

Physical and/or chemical treatments (e.g., reverse osmosis, chlorine, and ozone) are effective for removal of MCs, and several are regularly used in drinking water treatment facilities (Kumar et al., 2018). In addition, efforts have been made to develop alternative strategies for MC removal, e.g., the heterologous expression of the MlrA enzyme (Wang et al., 2017; Dexter et al., 2018), bioreactors with immobilized *Escherichia coli* expressing the MlrA enzyme (Dziga et al., 2014), or inoculated with MC degrading bacteria (Tsuji et al., 2006; Phujomjai et al., 2016; Kumar et al., 2019). Moreover, Dziga et al. (2018) have successfully combined a treatment with hydrogen peroxide, MC degrading bacteria, and a recombinant MlrA enzyme to accomplish complete MC removal.

Gravity-driven membrane (GDM) filtration systems are a robust, low-maintenance technology for the small-scale production of drinking water in communities without access to other types of water treatment (Peter-Varbanets et al., 2010; Pronk et al., 2019). After the initial reduction in flux due to biofilm formation on the membranes, a nearly constant and predictable amount of drinking water proportional to total membrane surface area can be produced by GDM systems over extended periods of time (Peter-Varbanets et al., 2017). Moreover, the microbial biofilms have distinct beneficial effects, e.g., they decrease the level of assimilable organic carbon in the filtrates, thereby delaying microbial regrowth (Derlon et al., 2014; Chomiak et al., 2015). Mature GDM biofilms can also be an effective means of removing MCs from toxic cyanobacteria in the feed water (Kohler et al., 2014). This degradation process could be rapidly induced even without extended prior exposure to the toxin, indicating that the original establishment of MC degraders in the biofilm communities was unrelated to this specific metabolic trait (Silva et al., 2018).

The aquatic system that provided the feed water for the above described GDM experiments (Lake Zurich) has a history of mass development of MC-containing cyanobacteria (Posch et al., 2012). Numerous MC degrading bacteria have been isolated from other aquatic habitats with regular cyanoHABs, such as Lake Taihu (Chen J. et al., 2010; Jiang et al., 2011; Yang et al., 2014; Zhang et al., 2017). Thus, it is likely that MC degraders were also present in the microbial assemblages of Lake Zurich that served as the inoculum for the GDM biofilm communities. MC removal possibly also occurs in GDM biofilm communities from source assemblages without prior exposure to toxic cyanobacteria. Numerous examples show the degradation of complex organic compounds by microbes enriched from diverse source communities at appropriate conditions (e.g., Cortes-Tolalpa et al., 2016), adding authority to Baas-Becking’s classical postulate that “everything is everywhere, but, the environment selects” (Baas Becking, 1934). However, while a particular metabolic activity such as biological MC removal might in principle occur in GDM biofilms produced from a wide range of microbial source communities, it is conceivable that this function will be carried out more or less efficiently depending on whether toxic cyanobacteria are present in the system that provides the feed water. Moreover, contrasting enrichment scenarios (i.e., on biofilms or in suspension) might amplify or diminish potential differences in MC degradation between such assemblages. Many planktonic habitats (and enrichment strategies for planktonic microbes) are characterized by turbulent mixing processes (Wüest and Lorke, 2003). By contrast, microbial biofilms are systems with steep vertical gradients and diffusion limitation (Stewart, 2003; Flemming et al., 2016). Microbes may drastically change their overall physiological state when alternating between their free-living and surface-attached life style (Costerton et al., 1995), and the biotic interactions within biofilms are arguably more complex than in the plankton (Moons et al., 2009), likely leading to contrasting patterns of competitive exclusion.

We tested if GDM biofilms fed with source water from a habitat with regular cyanoHABs would result in higher MC removal efficiency than from a habitat without such background. In addition, we hypothesized that potential differences might not merely be related to the presence or absence of MC-degrading bacteria in the source water, but might instead be rooted in community-related aspects of the biofilms. For this purpose, we studied two types of microbial consortia generated with water from Lake Zurich and from a stream without recorded occurrence of toxic cyanobacteria. On the one hand, we established biofilms within GDM filtration systems fed with water from the lake and stream, respectively. Second, we produced planktonic enrichments with pure MC at aerobic and anaerobic conditions.

## Experimental Procedures

### Gravity-Driven Membrane (GDM) Filtration Experiments

Two GDM experiments with comparable setups were conducted at two sites. The lake experiment (ExpL) was fed with water from Lake Zurich; it was conducted at the Limnological Station of the University of Zurich (location: N 47°19’ 13.24”, E 8°33’ 11.86”). The stream experiment (ExpS) was fed with water from the Chriesbach stream at the Swiss Federal Institute of Aquatic Science and Technology (N 47°24’ 16.30”, E 8°36’ 31.80”). Experiments were conducted in February/March 2017 over a period of 30 days.

**FIGURE 1:**
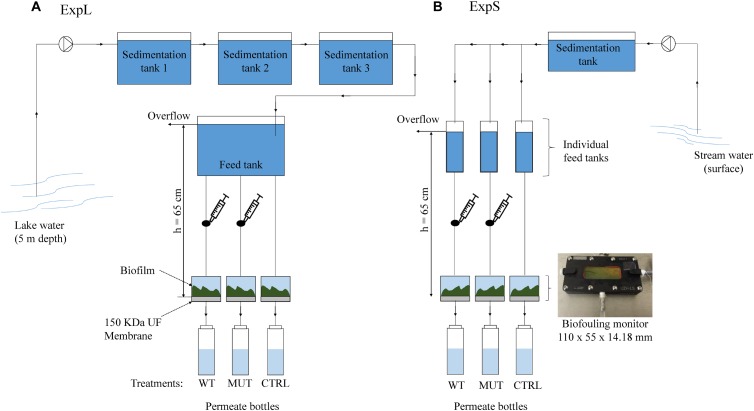
Experimental setup of **(A)** the lake experiment – ExpL and **(B)** the stream experiment – ExpS. Each experiment consisted of three treatments: WT – addition of *M. aeruginosa* biomass containing 1.7 μg MC mg^-1^ DW day^-1^; MUT – addition of biomass from the mutant *M. aeruginosa* strain that does not contain any MC (17 mg DW day^-1^); and CTRL – only the respective feed water. Each treatment had three biological replicates, except for the WT biomass treatment of ExpS which had five biological replicates. The syringe connection between the feed tank and the biofouling monitor was placed in order to add the correspondent biomass for the WT and MUT biomass treatment.

Both GDM systems were assembled in a comparable manner (Figure 1), with small differences due to local circumstances. ExpL featured three sedimentation tanks connected to a single 40 L feed tank (Figure 1A), while ExpS had one sedimentation tank that was connected to 11 individual feed tanks (Figure 1B). In ExpL, the lake water was directly pumped into the first sedimentation tank from 5 m depth, while in ExpS, the stream water was pumped from the surface of the Chriesbach stream. In both cases, the feed tanks had an overflow through which excess water could leave the system. For both experiments, the feed tank was connected to the biofouling monitors and placed 65 cm above the monitors to set the transmembrane pressure. Each biofouling monitor was equipped with a 150 kDa polyethersulfone ultrafiltration (UF) membrane (Microdyn Nadir, Wiesbaden, Germany). After passage through the UF membrane, the water was collected in permeate collection bottle. Syringe connections were assembled right above the influx of the biofouling monitor in order to supply the respective biomass without direct contact to the feed water (Figure 1). Before use, the UF membranes were soaked in 40% ethanol for 1.5 h and then rinsed overnight with sterile deionized water (Heffernan et al., 2013). The system between the UF membranes and the permeate bottles was sterilized; however, the bottles were replaced by new ones every 24 h in a non-sterile environment. The temperature in the feed water tank of both experiments was maintained at 20°C. Except for the permeate bottles, the whole system was kept in the dark. In both experiments, the transmembrane flux and MC removal were monitored every 24 h for a period of 30 days. Transmembrane flux was calculated from the water volume in the permeate bottles and the membrane area (19.95 cm^2^). Portions of 1 mL were taken daily from the permeate bottles and MC was quantified in these samples by HPLC-MS (see below). At the end of the experimental period, the biofilm thickness and structure was evaluated using optical coherence tomography (OCT) (model 930 nm Spectral Domain, Thorlabs GmbH, Germany), and mean thickness was determined by Matlab (MathWorks, United States) according to Derlon et al. (2013). Subsequently, the biofilms were collected and conserved at -20°C for later DNA extraction.

Each GDM experiment comprised three different treatments: the unamended treatment (CTRL) that only received water from the respective site, the wild-type treatment (WT biomass) that was additionally supplied with MC containing biomass from an axenic strain of *Microcystis aeruginosa* (see below), and the mutant treatment (MUT biomass) that was supplied with the same amount of MC-free biomass from an axenic mutant strain that does not produce MC (Dittmann et al., 1997). Both, the WT and MUT biomass treatments were supplied with the respective biomass (17 mg day^-1^ of dry cell weight [DW]) every 24 h. The WT biomass treatment received a MC (MC-LR and [D-Asp^3^] MC-LR) dose of 1.7 μg MC mg^-1^ DW day^-1^. ExpL included three replicates of each treatment (WT biomass, MUT biomass, and CTRL), while ExpS had five replicates for the WT biomass treatment and three replicates for the other two treatments (MUT biomass and CTRL). In ExpL, the conditions of the MUT biomass treatment were changed after day 18 in order to reproduce a previous observation about the MC degradation potential of such biofilms (Silva et al., 2018): the addition of MC-free biomass was replaced by addition of MC-containing biomass. This treatment was subsequently termed MUT+WT biomass.

### MC Retention Test

An additional GDM experiment was conducted with the purpose to understand to which extent the apparent initial MC removal in the GDM systems was caused by physicochemical retention on the membrane or adsorption to particles. A GDM system with three treatments (each with six replicates) was set up as described above, albeit using sterile bottles (10 L) in place of a feed tank. These bottles were filled (i) with sterile deionized water, (ii) with pasteurized lake water (80°C, 7 min), and (iii) with raw lake water. Each treatment (sterile, pasteurized, and raw water) was initially supplied with MC-containing biomass from *M. aeruginosa* (1.7 μg MC mg^-1^ DW day^-1^). After 22 h, the amount of MC that had passed through the membrane into the permeate bottle was quantified via HPLC-MS (see below). The proportion of MC retained on the membrane was calculated from the difference between the total MC input and the MC recovered in the permeate bottle.

### Production of Cyanobacterial Biomass and MC Quantification

The cultures of wild type *M. aeruginosa* PCC7806 and a MC-deficient mutant strain (Dittmann et al., 1997) that were used to supplement the GDM biofilms were grown axenically in a medium developed for cyanobacteria cultivation (Jüttner et al., 1983) without addition of chloramphenicol, at constant temperature and light (20°C, 3.5 μmol quanta m^-2^ s^-1^). Axenic growth was repeatedly verified by microscopic inspection of the cultures after staining with 4′,6-diamidino-2-phenylindole (DAPI). The wild type strain produces two MC variants, MC-LR and [D-Asp^3^] MC-LR. The strains were inoculated into replicate 100 mL culture flasks in order to produce sufficient material for the whole experiment, i.e., a total of 4 and 3 L for the wild type and mutant strains, respectively. Biomass was harvested from dense cultures (after ca. 3 weeks of growth) of the two strains, concentrated by centrifugation and then frozen at -20°C in 50 mL aliquots. Three freeze-thaw cycles were performed to destroy cyanobacterial cells and to release the MC from the wild type strain. A subsample was taken to determine dry cell weight and the rest was reserved to supplement the GDM systems. MC concentration in the biomass of the wild type strain was quantified by HPLC-MS, which also served to confirm the absence of MC in biomass from the mutant strain.

Microcystin was quantified as described previously (Silva et al., 2018). Briefly, samples were first filtered (0.2 μm polyethersulfone filter) and then diluted with pure methanol (final concentration 70%). An HPLC 1260 Infinity series system (Agilent Technologies) interfaced with an API 500 triple quadrupole mass spectrometry system (AB Sciex) were used to separate and detect MC. The limit of detection was 0.5 μg L^-1^ and the limit of quantification 1 μg L^-1^ for both MC (MC-LR and [D-Asp^3^] MC-LR). The software Analyst (version 1.6.1, AB Sciex) and MultiQuant (version 2.1, AB Sciex) were used for data acquisition and the quantification of two MC variants: MC-LR (*m/z* 995.5) and [D-Asp^3^] MC-LR (*m/z* 981.5), respectively.

### Enrichments Experiment With Pure MC

Enrichments of lake and stream water samples with pure MC-LR were performed to independently investigate the occurrence of (planktonic) MC degraders in both habitats and to obtain additional information about their physiology. Serum bottles (100 mL) were filled either with water from Lake Zurich or from the Chriesbach stream. Three of the six replicate bottles per site were maintained under aerobic and anaerobic conditions, respectively. The aerobic incubations were continuously supplied with sterile (0.2 μm filtered) air. The anaerobic enrichments were maintained inside an anaerobic bench (Anaerobic System Typ 1029, Brouwer AG, Switzerland). The bottles were then spiked with pure MC-LR (purity ≥ 95%, Cayman Chemical, United States) achieving a final concentration of 20 μg L^-1^ and incubated in the dark at a constant temperature of 20°C. Samples were collected regularly over a period of 15 days and MC-LR was quantified via HPLC-MS as described above. At the end of the experiment, the enriched biomass (altogether ca. 80 mL) was collected and preserved at -20°C for DNA extraction.

### DNA Extraction, 16S rRNA Gene Sequencing, and Taxonomic Assignment

DNA was extracted at the end of each experiment (GDM: day 30, enrichments: day 15) from 20 whole membranes of the GDM biofilms and from the remaining volume (∼80 mL) of 12 samples of the enrichment experiment using the DNeasy PowerBiofilm Kit (Qiagen, Germany). The extraction was largely performed according to the manufacturer’s specifications, except for the inhibitors removal step that was extended to 1 hour. The purified DNA was stored at -20°C in 10 mM Tris buffer for further analysis. DNA was amplified with the primer pair 799F-1115R that exclude chloroplasts/cyanobacteria (Chelius and Triplett, 2001; Redford et al., 2010); the reverse primer was modified according to Silva et al. (2018). Partial 16S rRNA gene sequences were obtained using an Illumina MiSeq platform (LGC Genomics, Germany). Raw data processing, the definition of operational taxonomic units (OTUs), and their taxonomic assignment were performed by an in-house pipeline as described previously (Silva et al., 2018). Raw sequencing data have been deposited on the Sequence Read Archive of NCBI under the project number PRJNA529182.

### Statistical Analyses

All statistical tests were performed in R (version 3.3.2). Prior to the analyses of the sequencing data, two of the five replicates of the WT treatment from ExpS were randomly excluded to obtain an equal number of replicates for all treatments. The remaining data set was normalized to the sample with the lowest total number of sequence reads as described previously (Silva et al., 2018).

The samples from the GDM experiment were average linkage clustered based on Bray–Curtis dissimilarity scores. The stability of the main branches was tested by cluster-wise stability tests (1000 bootstrap interactions), and significant differences between groups were discovered using similarity profile analyses (α = 0.001). For this, the R packages *vegan*, *clustsig*, and *fpc* were used (Oksanen et al., 2011; Whitaker and Christman, 2014; Hennig, 2015).

For a co-occurrence network of OTUs with the 18 samples of the GDM experiments (ExpL and ExpS), Spearman’s rank correlations were calculated between pairs of OTUs that were present in at least three samples. The *p*-values were adjusted with the Benjamini–Hochberg method for multiple testing correction (Hochberg and Benjamini, 1995). These calculations were done using the *Hmisc* R package (Harrell, 2016). Only OTU pairs with statistically significant (*p* < 0.01) and robust Spearman’s correlation (ρ ≥ 0.6 or ρ ≤-0.6) were considered as a valid positive or negative co-occurrence event. The data were exported in GML format file using the R package *igraph* (Csardi and Nepusz, 2006). Visualization and analysis (modularity, diameter, average path length, density, and betweenness centrality) were done using the program *Gephi* (Brandes, 2001; Blondel et al., 2008; Bastian et al., 2009).

**FIGURE 2:**
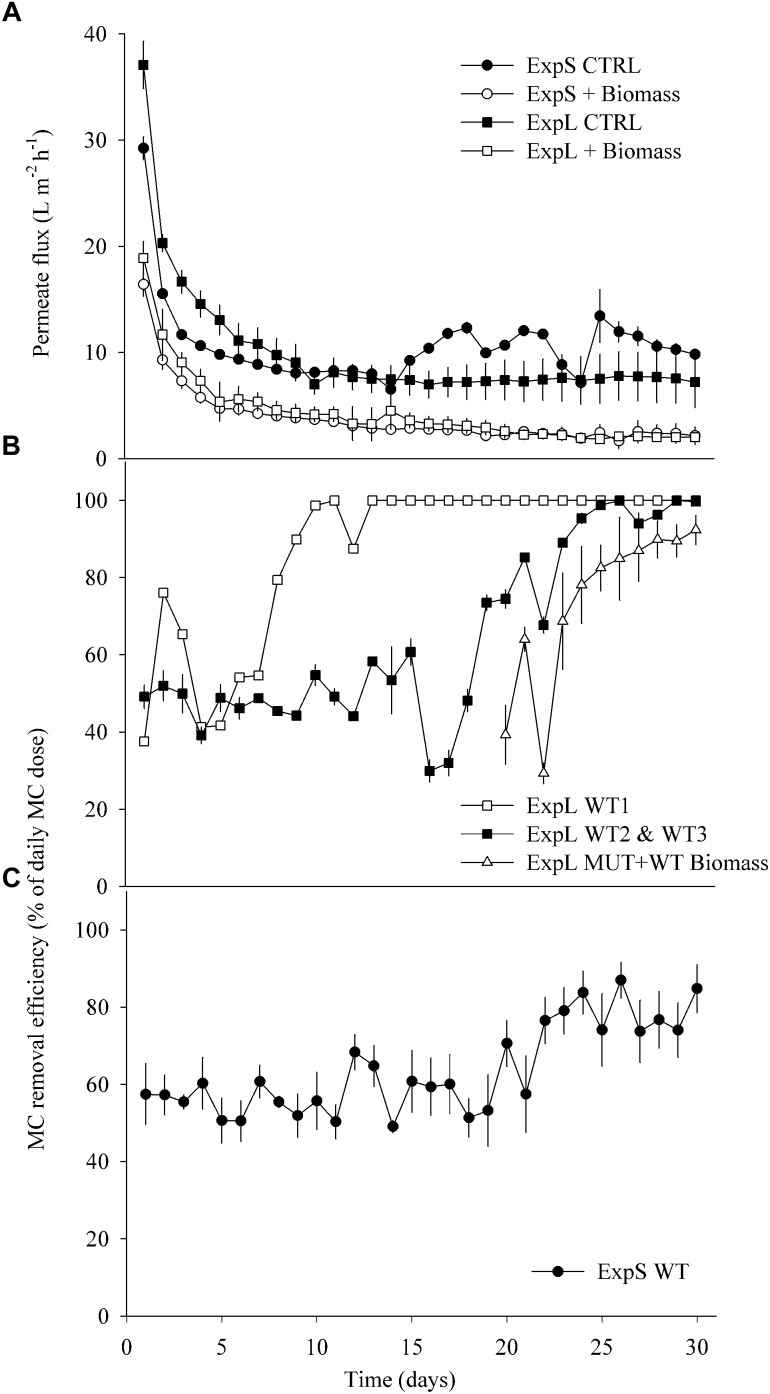
**(A)** Average permeate flux in ExpL and ExpS. The label “+Biomass” treatment refers to the average of the WT biomass and MUT (+WT biomass) treatments and the label “CTRL” to the treatments that received only lake or stream water. **(B)** Microcystin removal efficiency in ExpL. Note that the MUT+WT biomass treatment was supplemented with MC-free biomass until day 18 and with MC-containing biomass thereafter. WT 1, 2, and 3 refer to the three biological replicates of the WT treatment. **(C)** Microcystin removal efficiency in ExpS. Data are depicted as means ± one standard deviation.

**FIGURE 3:**
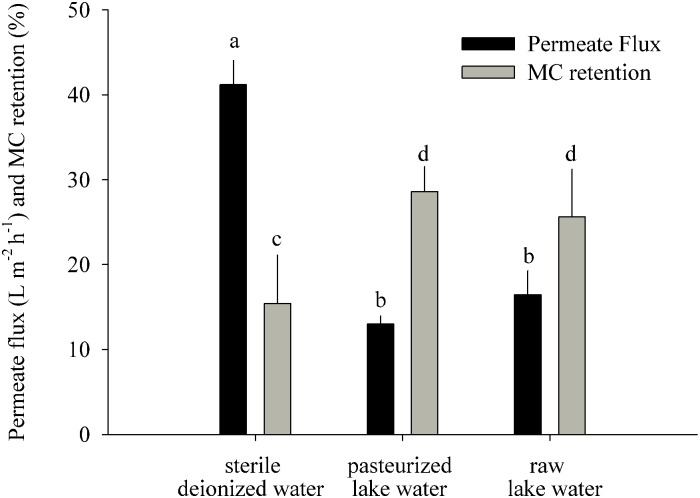
MC retention test: permeate flux and MC retention measured 22 h after addition of MC-containing biomass to the GDM system (means ± one standard deviation). Letters denote treatments that are significantly different for the permeate flux and for the MC retention, respectively (ANOVA followed by Tukey’s HSD tests, *p* < 0.001–0.05).

Differences between data sets were tested for significance by Student’s *t*-tests or analyses of variance (ANOVA) followed by Tukey’s honestly significant difference (HSD) test. All proportional data were *logit* transformed (Warton and Hui, 2011). We tested if there were differences (i) in MC retention/removal between ExpL and ExpS during the first and last week of the experiments, as well as over the complete experimental period (repeated measures ANOVA) (Figure 2B,C); (ii) in MC retention and permeate flux between the treatments raw lake water, pasteurized lake water, and deionized water (Figure 3); (iii) in the degree number (i.e., the number of links between individual OTUs or nodes) between the 5 modules of the co-occurrence network (Figure 5C); (iv) in the proportions of OTUs that occurred at one or both experimental sites and/or treatments; and (v) in the proportions of OTUs that occurred in only one or in all three biological replicates of a particular treatment (Table 2).

## Results

### GDM Biofilm Properties

The four biomass-amended treatments of both experiments (ExpL and ExpS) behaved similarly in terms of flux decline, by approximately 70% during the first week of operation (Figure 2A). Afterward, all the four biomass-amended treatments stabilized around 2.8 ± 1 L m^-2^ h^-1^. In ExpL, the CTRL treatment stabilized at 7.5 ± 0.3 L m^-2^ h^-1^, after 80% of flux decline over the first 9 days of operation. In ExpS, this treatment had a small period of stable flux with 8 ± 0.3 L m^-2^ h^-1^, but it exhibited irregularities after 13 days of operation, varying between 6 and 13 L m^-2^ h^-1^ (Figure 2A).

At the end of the experiment, the biofilm thickness of the CTRL treatments was on average 52 ± 7 and 105 ± 19 μm for ExpL and ExpS, respectively. For the biomass-amended treatments, it was not possible to exactly determine the biofilm thickness because in many replicates the biofouling monitor was completely filled with biofilm/fouling cake (∼1 mm thickness). In terms of horizontal structure, the CTRL biofilms of ExpS exhibited higher heterogeneity compared with ExpL (Supplementary Figure S1).

In ExpL, one of the three replicates of the WT treatment reached 100% of MC removal efficiency after 10 days of operation, while the other two replicates required 25 days until they completely removed the newly added MC (Figure 2B). As observed previously (Silva et al., 2018), the addition of toxic biomass to the MUT treatment led to a rapid upshift of MC removal in these biofilms, to 69 ± 16% after 4 days and to 92 ± 6% after 10 days. The MC removal in each biological replicate of ExpS was generally rather unstable (data not shown). However, the average of all replicates resulted in a reliable MC removal efficiency (Figure 2C): it remained unaltered around 57 ± 6% during the first 20 days of operation, subsequently increased by approximately 20% and fluctuated between 58 ± 10 and 85 ± 6% until the end of the experiment. During the first week of GDM operation, MC removal was significantly lower in ExpL than in ExpS, and significantly higher during the last week, as indicated by both, Repeated Measures ANOVA and Student’s *t*-tests (*n* = 7, *p* < 0.05). MC removal over the entire period was significantly higher in ExpL (Repeated Measures ANOVA, *n* = 30, *p* < 0.001).

### Microcystin Retention on the GDM Membrane

This experiment was performed to understand how much of the initial MC removal was caused by its abiotic retention on the membrane (Figure 3). The most likely physicochemical processes involved in initial MC retention were the adsorption of MC to the membrane surface and to particles introduced by the influent water and captured on the membrane. The sterile water treatment had a significantly higher permeate flux (41 ± 3 L m^-2^ h^-1^) than the pasteurized (13 ± 1 L m^-2^ h^-1^) and raw lake water (16 ± 3 L m^-2^ h^-1^) treatments (Figure 3) (ANOVA, *n* = 6, *p* < 0.001) and significantly lower MC retention (15 ± 6%) than the pasteurized (29 ± 3%) and raw water (26 ± 5%) treatments (ANOVA, *n* = 6, *p* < 0.05).

### Community Diversity of GDM Lake and Stream Biofilms

After normalization to the smallest sample (4904 reads per sample), the sequence data set comprised 1941 OTUs. *Proteobacteria* constituted 62% of all reads (52% of OTUs, Supplementary Table S1), followed by *Bacteroidetes* (11 and 14% of reads and OTUs, respectively), *Actinobacteria* (7 and 6%), and *Firmicutes* (2.4 and 4%). Several other phyla also formed > 1% of total reads (*Parcubacteria*, *Acidobacteria*, *Chloroflexi*, *Spirochaetae*, *Deinococcus*, *Fibrobacteres*, and *Chlamydiae*). There was a significant difference in the relative abundances (read numbers) of the different phyla between sites (paired *t*-test, *n* = 13, *p* < 0.01), but not between treatments (Supplementary Table S1).

#### Differences Between Treatments

In general, the largest OTUs (most abundant in terms of read numbers) were not exclusive to a particular treatment: <20% of treatment-independent OTUs formed >75% of total reads. However, 11 of these 20 largest OTUs (Table 1) were significantly (>8 times) more abundant in the biomass-amended treatments than in the CTRL. In turn, the CTRL biofilms harbored significantly (>3 times) larger proportions of treatment-exclusive OTUs (35%), but these OTUs together only represented <10% of total reads.

**Table 1:** Top 20 most abundant OTUs present in the ExpL and ExpS.

Proportion of total	Ratio	Ratio	AccNR (% identity)	Taxonomy
sequences (%)	ExpL:ExpS	Biomass:CTRL	Taxonomy (genus)	(phylum; order)
6.0	1.7	9.8	JF166723 (100%)	Proteobacteria
			*Pseudoxanthomonas* sp.	Xanthomonadales
5.9	19.3	8.8	KM083544 (100%)	Proteobacteria
			*Lysobacter* sp.	Xanthomonadales
4.0	0.003	253.1	GU214129 (100%)	Deinococcus-Thermus
			*Deinococcus* sp. 1	Deinococcales
3.9	0.7	0.3	AB515715 (100%)	Proteobacteria
			*Rhodobacter* sp. 1	Rhodobacterales
2.3	0.1	18.4	AB769197 (100%)	Proteobacteria
			*Ideonella* sp.	Burkholderiales
2.0	5.2	46.5	KJ808529 (100%)	Proteobacteria
			*Thermomonas* sp.	Xanthomonadales
1.8	5.5	1.0	FJ612179 (100%)	Proteobacteria
			*Rhodobacter* sp. 2	Rhodobacterales
1.5	1.4	1.3	AJ784892 (100%)	Bacteroidetes
			*Haliscomenobacter hydrossis*	Sphingobacteriales
1.4	9.3	0.0	DQ664243 (100%)	Proteobacteria
			*Sphingopyxis* sp.	Sphingomonadales
1.3	0.01	290.3	JQ511861 (100%)	Deinococcus-Thermus
			*Deinococcus* sp. 2	Deinococcales
1.3	0.4	82.1	FJ516972 (99%)	Fibrobacteres
			uncultured bacteria	Chitinivibrionales
1.3	^∗^	^∗^	LN571172 (100%)	Parcubacteria
			*uncultured bacteria*	*Candidatus Magasanikbacteria*
1.2	^∗∗^	^∗∗^	LN573232 (100%)	Actinobacteria
			*Mycobacterium* sp.	Corynebacteriales
1.0	0.003	0.005	KT905668 (100%)	Actinobacteria
			*Rhodococcus* sp.	Corynebacteriales
1.0	^∗∗^	1.0	KF697567 (100%)	Proteobacteria
			*Haliangium* sp.	Myxococcales
1.0	0.7	31.8	JQ072374 (100%)	Proteobacteria
			*Inhella* sp.	Burkholderiales
0.9	0.8	0.5	KT029151 (100%)	Proteobacteria
			*Hydrogenophaga* sp.	Burkholderiales
0.9	64.8	196.8	AF534435 (100%)	Bacteroidetes
			uncultured bacteria	Sphingobacteriales
0.9	1.6	9.8	KT182550 (100%)	Proteobacteria
			*Arenimonas* sp.	Xanthomonadales
0.8	0.8	12.1	HG530247 (100%)	Proteobacteria
			*Paucibacter toxinivorans*	Burkholderiales


*^∗^Present in one replicate of ExpL.*

*^∗∗^Present in ExpS and less than three reads in the ExpL.*

*The OTUs overrepresented in the biomass-amended compared to the CTRL treatments were considered to be biomass responders.*

#### Differences Between Sites

Operational taxonomic unit richness was 1422 and 1069 in ExpS and ExpL, respectively, and significantly higher fraction of OTUs were exclusively found in the stream biofilms (45 vs. 27%). Eight of the top 20 most abundant OTUs (Table 1) were significantly more abundant in ExpL than in ExpS, whereas two OTUs were highly abundant in ExpS but had <3 reads in ExpL. The fractions and read numbers of OTUs that were common to both sites were significantly higher in the WT biomass than in the CTRL biofilms (69 vs. 48% and 90 vs. 62%, respectively, Student’s *t*-tests, *n* = 6, *p* < 0.01). By contrast, the CTRL communities were a significantly better niche for site-specific OTUs (18 vs. 11%, Student’s *t*-test, *n* = 12, *p* < 0.001) that also had significantly more reads (8 vs. 2%, Student’s *t*-test, *n* = 12, *p* < 0.001). Thus, the addition of biomass generally reduced the site-specific differences between the biofilm communities.

#### Differences Between Biological Replicates

Operational taxonomic unitss were defined as being either “*stochastic*” or “*deterministic*” community members if they were present in only one or in all three biological replicates of a particular treatment. On average, only <50% of all OTUs were “*deterministic*” colonizers of all replicates of a treatment, whereas one-third fell into the “*stochastic*” category (Table 2). A significantly larger fraction of these “*stochastic*” OTUs were only found at one of the two experimental sites (17 vs. 12%). By contrast, significantly higher proportions of the “*deterministic*” OTUs (31 vs. 12%) were present at both sites (Student’s *t*-tests, *n* = 12, *p* < 0.05). Moreover, the proportions of “*deterministic*” OTUs that occurred at both sites and in every replicate were significantly higher in the WT than in the CTRL treatments (Student’s *t*-tests, *n* = 6, *p* < 0.001), indicating that the addition of biomass favored a more reproducible colonization by cosmopolitan bacterial genotypes.

**Table 2:** Abundances of OTUs in the different treatments (means ± one standard deviation) and proportions of OTUs (means ± one standard error) that were only present in one biological replicate (“stochastic”) or in all the three biological replicates (“deterministic”).

Treatment (*n* OTUs)	Stochastic		Deterministic	
		
	Shared	Unique	Shared	Unique
Lake WT (284 ± 40)	0.13 (±0.03)	0.13 (±0.02)	0.40 (±0.06)	0.06 (±0.009)
Stream WT (365 ± 30)	0.11 (±0.01)	0.16 (±0.03)	0.37 (±0.03)	0.09 (±0.007)
Lake CTRL (391 ± 74)	0.18 (±0.03)	0.2 (±0.03)	0.24 (±0.05)	0.11 (±0.02)
Stream CTRL (545 ± 48)	0.08 (±0.01)	0.19 (±0.04)	0.22 (±0.02)	0.23 (±0.02)
Average WT	0.12 (±0.02)	0.15 (±0.03)	0.39 (±0.28)^c^	0.07 (±0.02)
Average CTRL	0.13 (±0.05)	0.2 (±0.03)	0.23 (±0.04)^c^	0.17 (±0.07)
Total (average)	0.12 (±0.04)^a^	0.17 (±0.04)^a^	0.31 (±0.09)^b^	0.12 (±0.07)^b^


*The terms “shared” and “unique” refer to OTUs that either occurred at both sites (lake and stream) or were exclusive to one site. Superscripts denote treatments that were tested for differences by Student’s *t*-tests (a, b: *n* = 12, *p* < 0.05; c: *n* = 6, *p* < 0.001).*

#### Dissimilarity Analysis

**FIGURE 4:**
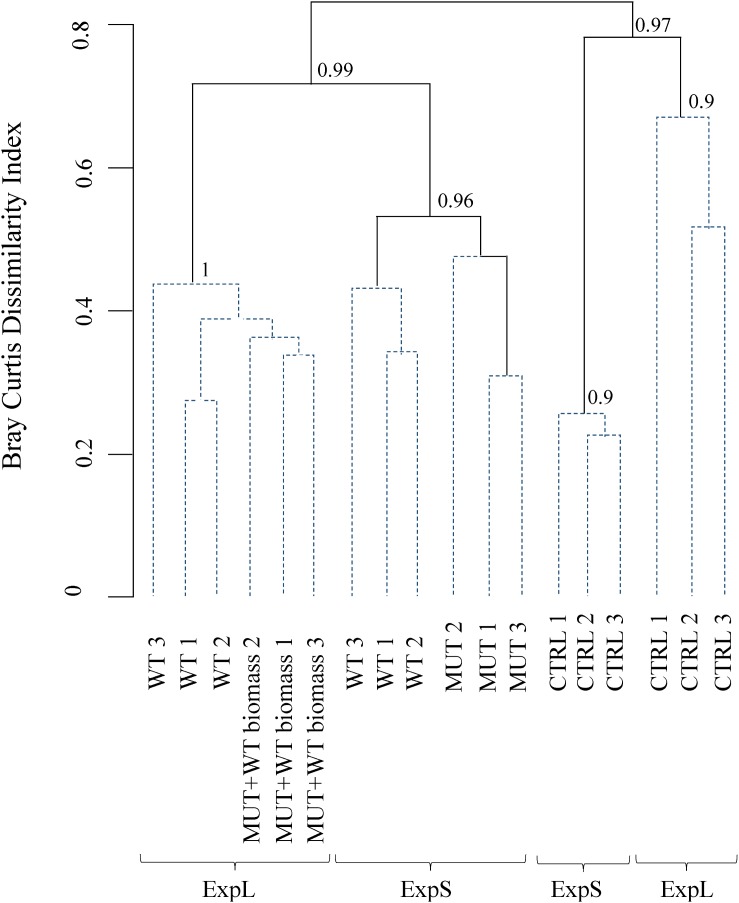
Clustering of microbial communities from both experiments according to Bray–Curtis dissimilarity. Similarity profile analysis identified five significantly different groups (solid lines). Biological replicates were not significant different (α = 0.001, dashed lines) from each other. The cluster stability was tested by bootstrapping (1000 times interaction); bootstrap values are indicated at the branching points.

When sequence data were clustered according to Bray–Curtis dissimilarity, the main separation between the communities was according to treatment rather than site (Figure 4). Bootstrap analysis indicated high stability of the four major branchings. Within the two branches of biomass-amended samples, one cluster included all samples of ExpL, and the other two clusters (of approximately 40% similarity) were the WT and MUT treatments of ExpS. The CTRL treatments from both sites fell within the second major branch, with only 20% similarity between each other. A similarity profile analysis revealed five statistically significant groups of samples: (i) the biomass-amended treatments of ExpL, (ii) the WT treatment of ExpS, (iii) the MUT treatment of ExpS, (iv) the CTRL treatment of ExpL, and (v) the CTRL treatment of ExpS. None of the biological replicates were significantly different (α = 0.001) to each other, and neither were the two biomass-amended treatments of ExpL (WT and MUT+WT biomass).

### Microbial Co-occurrence Network

**FIGURE 5:**
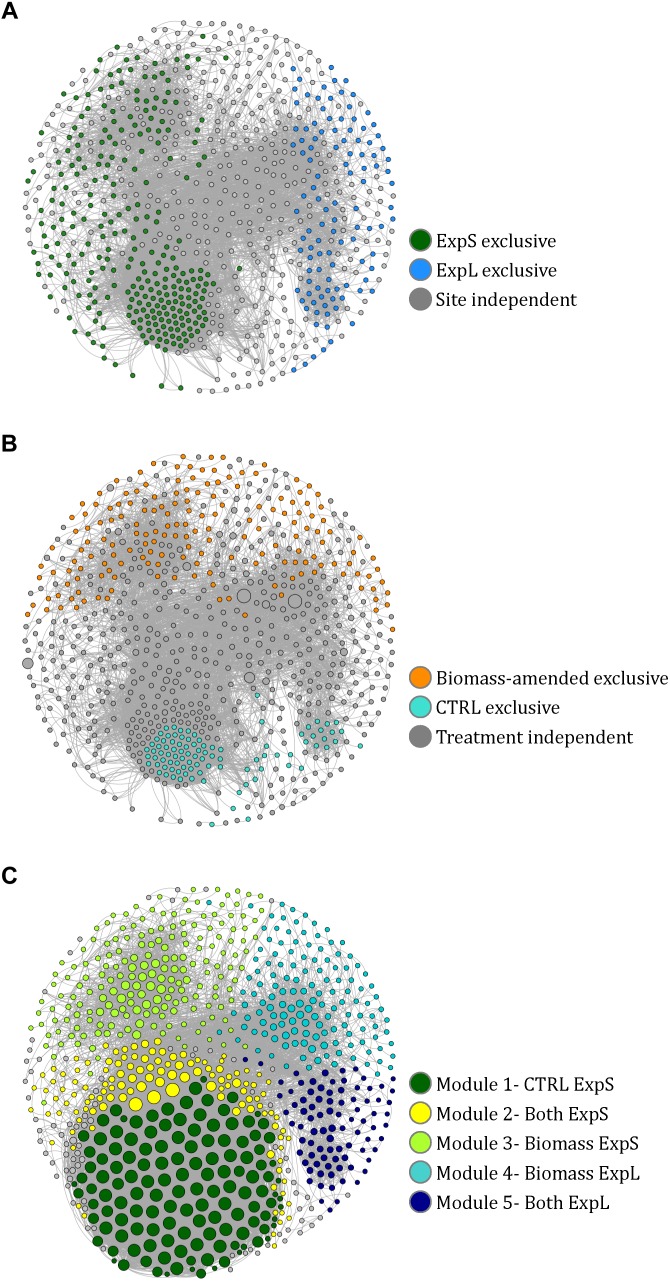
Co-occurrence network of 757 nodes (i.e., significantly correlated OTUs) from the GDM biofilm communities. **(A)** Nodes colored according to their exclusive occurrence at one site. **(B)** Nodes colored according to their exclusive occurrence in particular treatments and sized according to read numbers. **(C)** Nodes colored according to modularity class and sized according to degree (i.e., number of links to other nodes). The naming of the modules in the legend is based on the observations in **A** and **B**.

A co-occurrence network was constructed to assess to which extend the individual biofilm communities were composed of the same sets of bacteria at the two sites and within different treatments. The complete network of OTUs from both sites and all treatments comprised 757 nodes (39% of all OTUs and 91% of all reads) linked by 12,198 edges (Figure 5). Ninety-five percent of the network consisted of positive correlations, and negatively correlated OTUs predominantly occurred in different treatments or at different sites. The network had a diameter of 16, an average path length of 5.6, a density of 0.04, and a modularity of 0.5 (Brandes, 2001; Blondel et al., 2008). A more detailed description of network properties and of OTUs specific to the different sites and treatments is given in Supplementary Table S2.

Fifteen distinct modules (coherent sub-networks) were identified based on edge weights and randomization (Blondel et al., 2008). The five major modules represented 91% of all reads in the network (Figure 5C). We could assign these modules to experimental sites and treatments by the location of those OTUs that were only present at one site or in one treatment (Supplementary Table S3): All OTUs that only occurred in ExpS fell into modules 1, 2, and 3 (named ExpS-CTRL, ExpS-Both, and ExpS-Biomass, respectively, Supplementary Table S3), while the modules 4 and 5 (named ExpL-Biomass and ExpL-Both, respectively, Supplementary Table S3) harbored all OTUs that were exclusive to ExpL (Figure 5A). Moreover, modules 3 and 4 included all the exclusive OTUs from the biomass-amended treatments, while the modules 1, 2, and 5 included all the OTUs that were specific to the CTRL treatments (Figure 5B). The ExpS-specific modules 1–3 had significantly higher average degree number (i.e., the number of OTUs that significantly co-occurred with each other) than the ExpL-specific modules 4 and 5 (9660 vs. 3665), indicating a generally more synchronized occurrence of OTUs within ExpS communities (Student’s *t*-test, *n* = 226, *p* < 0.001, Figure 5C).

### Communities Enriched With Pure Toxin

**FIGURE 6:**
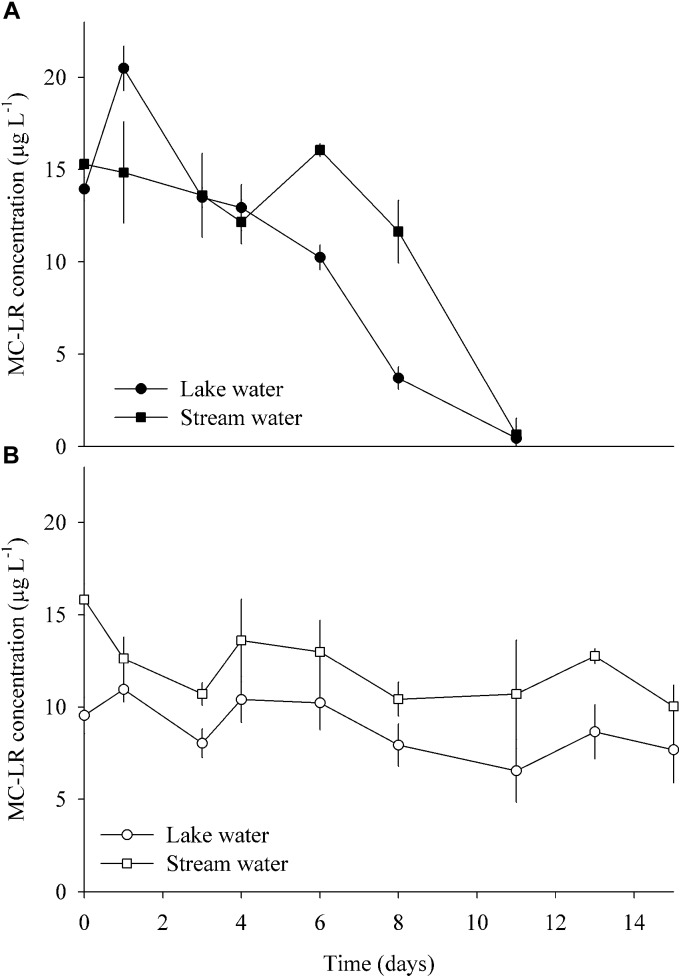
MC degradation in enrichment cultures amended with pure MC, using water from the lake and stream, respectively, and incubated under **(A)** anaerobic and **(B)** aerobic conditions (means ± one standard deviation of three replicate incubations).

Enrichment cultures from both sites amended with pure MC completely consumed the toxin after 11 days of incubation at anaerobic conditions (Figure 6A). By contrast, MC concentrations only decreased by 20 and 36% after 15 days of aerobic incubations of lake and stream water, respectively. Following normalization to the smallest sample (19,029 reads), there were 589 OTUs in the enrichment cultures. About one-quarter of these OTUs (153) were only present in the lake water enrichments, while approximately half (242) were exclusive to the stream water enrichments. Seventy-four percent of all OTUs (13% of reads) were only found in the anaerobic incubations, and 12% (3% of reads) only in the aerobic ones. The 10 most abundant OTUs (>5000 reads, Supplementary Table S4) represented 65% of the total amount of reads.

Thirty-eight percent of all OTUs from the enrichment experiment were also present in the GDM network, including three of the five most abundant enriched OTUs (*Limnobacter* sp., *Acinetobacter* sp., and *Brevundimonas vesicularis*). Contrariwise, the two most abundant OTUs in the GDM network (*Pseudoxanthomonas* and *Lysobacter*) were also found in all samples of the enrichment cultures. Two OTUs related to known MC degraders were present in both, the enrichments and the GDM network: *Paucibacter toxinivorans* (in all enrichment samples) and *Stenotrophomonas rhizophila* (in 10 of 12 enrichment samples).

## Discussion

### Physical Properties of Lake and Stream Water Fed GDM Biofilms

As observed previously, permeate flux in GDM systems stabilized after approximately 1 week of operation (Peter-Varbanets et al., 2010). Biomass addition led to twice as much hydraulic resistance and >3 times thicker biofilms (Figure 2A and Supplementary Figure S1), resulting in lower flux (McCarthy et al., 2002; Kohler et al., 2014; Silva et al., 2018). Permeate flux and biofilm thickness of the biomass-amended biofilms in ExpL and ExpS were similar, suggesting that their hydraulic properties were neither affected by the respective characteristics of the lake and stream water influx nor by the slight differences in setup. By contrast, the permeate flux of the two CTRL treatments was markedly different. It is conceivable that the three sedimentation tanks of ExpL allowed for higher particle removal than the single tank of ExpS (Figure 1). Also, the raw water for ExpL was pumped into the system from 5 m depth while it came from the surface of the stream for ExpS, which might have influenced the respective loads of inorganic particles and consequently flux (Chomiak et al., 2014). On the other hand, CTRL biofilms in ExpS were on average twice as thick as in ExpL (Supplementary Figure S1), but had higher and unstable flux rates (Figure 2A). This might be an indication for the activity of larger organisms (Supplementary Figure S1) feeding on the stream water biofilms (Derlon et al., 2012, 2013).

Our short-term experiments suggested that the partial removal of MC from filtrates during the first week of GDM operation was mainly due to retention of the toxin by abiotic processes (Figure 3). About two-thirds of MC in cyanobacterial cells are bound to protein (Zilliges et al., 2011; Wei et al., 2016), and a large proportion of cell fragments is likely physically captured by the membrane. In addition, the passive adsorption to particles or exo-polymeric substances may also play a role, as suggested by significantly higher MC retention in pasteurized lake water samples than in sterile deionized water (Figure 3). A higher loading with inorganic particles in the stream water biofilms might explain the significantly higher levels of initial MC retention in ExpS (Figure 2B,C) at comparable flux rates (Figure 2A) (Morris et al., 2000). Previous GDM experiments with feed water from Lake Zurich showed somewhat lower initial MC retention levels (Kohler et al., 2014; Silva et al., 2018) possibly due to seasonal factors, e.g., the higher abundances of bacteria and other microbes during spring (Salcher et al., 2011; Eckert et al., 2012).

### Higher MC Removal in Lake Water Generated Biofilms

Microcystin removal during the last week of GDM operation was a combination of abiotic retention of the toxin on the membrane filters (Figure 2B,C, 3) and its biological degradation. Microbial MC consumption could again be “primed” by addition of non-toxic cyanobacterial biomass in ExpL biofilms (Silva et al., 2018), suggesting that it was a facultative trait of bacteria with a broader metabolic potential, and that their initial establishment in the biofilm communities was unrelated to their ability to consume the toxin. This might be important for understanding the relationship between biofilm function and community structure.

Interestingly, the performance of ExpL biofilms with respect to biological MC removal was strikingly different between biological replicates (Figure 2B) without corresponding differences in permeate flux (Figure 2A). This might be interpreted in the context of stochastic community assembly processes, i.e., communities shaped by immigration and random processes rather than by optimal fitness of community members for a particular environmental niche (Zhou and Ning, 2017): experiments in fermenters (Zhou et al., 2013) and with lake water enrichments (Langenheder et al., 2006) have shown that quasi-identical growth conditions may lead to microbial communities that differ both in composition and functional properties. Notwithstanding these initial differences between replicates, MC removal efficiency in ExpL was significantly higher during the late phase even though permeate flux (Figure 2) and biofilm thickness were indistinguishable: while lake water biofilms increased MC retention by roughly 50% between the first and the last week and eventually completely removed the daily doses of freshly added MC, the biotic degradation in stream water biofilms led to final MC removal levels that were only approximately 20% above the initial, abiotic (Figure 2) “background.” Consequently, the average MC removal rate was significantly lower in ExpS (40 ± 4.3 μg L^-1^ day^-1^) than in ExpL (49 ± 2.5 μg L^-1^ day^-1^, mean ± standard deviation, *n* = 3). MC removal rates comparable to those of ExpL were also reported from enrichments of lake water featuring prior blooms of toxic cyanobacteria (Li et al., 2016). The dominant phytoplankton species in Lake Zurich is the MC-containing cyanobacterium *Planktothrix rubescens* (Posch et al., 2012), whereas there is no recorded history of blooms of toxic cyanobacteria in Chriesbach stream.

The biofilm communities of ExpL and ExpS significantly differed in composition even at the level of phyla (Supplementary Table S1). Yet the lower MC removal in ExpS biofilms cannot be attributed to the absence of equally efficient MC degrading microorganisms in the stream feed water: bacteria from Chriesbach enrichment cultures consumed MC to similar final concentrations as bacteria from Lake Zurich (Figure 6A). Thus, lower MC removal by stream water biofilms must have been due to the less favorable (biotic and abiotic) conditions for the growth of these degraders. The differences in MC removal in ExpL and ExpS might thus reflect that the process is not indigenous in stream water, but that nevertheless, in analogy with Baas Becking’s proposition (Baas Becking, 1934), “every functional potential is everywhere.” A widespread potential for MC degradation has already been noted by Jones and Orr (1994).

Only six of the most abundant genotypes from the biofilms (Table 1) and the enrichment experiment (Supplementary Table S4), have been associated with MC degradation previously: *P. toxinivorans*, *Sphingopyxis* sp., *Rhodococcus* sp., *Acinetobacter* sp., *Methylotenera* sp., and *Pseudomonas* sp. (Li et al., 2017). Involvement of *Paucibacter* in MC degradation was suggested by its >10-fold higher read numbers in the biomass-amended GDM treatments (Table 1); however, it only occurred in aerobic enrichments, where little MC degradation was observed (Figure 6B). *Sphingopyxis* and *Rhodococcus* were mainly enriched in the GDM CTRL treatments (Table 1), excluding them as potential MC degraders, which is in line with previous observations by Li et al. (2016). Our results also support another conclusion of that study, i.e., the putative role of *Methylotenera* sp. in MC degradation (Supplementary Table S4).

### Addition of Biomass Selects for Widely Spread Bacterial Taxa

Bray–Curtis analyses (Figure 4) indicated that the dissimilarity between communities was most strongly driven by the treatments. This was likely related to the occurrence of OTUs at one or at both experimental sites: significantly more bacteria of the WT than of the CTRL biofilms were present in both ExpL and ExpS biofilms. A large fraction of these OTUs, moreover, was found in all three replicates of a particular treatment type (Table 2), suggesting environmental filtering (Cao et al., 2016) as the predominant selection mechanism in biomass-amended biofilms. Indeed, the most abundant of these genotypes were from widely spread bacterial genera: *Pseudoxanthomonas* spp. have been isolated from sludge, soils (Thierry et al., 2004), hot spring (Chen et al., 2002), and compost (Weon et al., 2006) environments, *Lysobacter* spp. have been found in soil, the rhizosphere, and freshwater (Reichnbach, 2006), and *Arenimonas* spp. occur in diverse habitats such as oil-contaminated soil (Young et al., 2007), the seashore (Kwon et al., 2007), an iron mine (Chen et al., 2012), or a eutrophic reservoir (Huy et al., 2013). By contrast, the CTRL treatment harbored significantly more OTUs that only occurred at a single experimental site and/or in only a single biological replicate (Table 2), indicating that these communities were more stochastically assembled from the local species pools. The two most abundant of these “*site-specific*” genotypes in ExpL biofilms were from groups strictly associated with freshwaters: the novel candidate phylum Parcubacteria (Brown et al., 2015), and *Actinobacteria* from the ac1 lineage (Newton et al., 2007).

Thus, the richer growth conditions after addition of biomass replaced the “indigenous” (i.e., site-specific) biofilm flora by a more widely spread set of bacteria that, moreover, colonized the replicate biofilms in a more reproducible manner. This change was accompanied by a loss of overall genotypic diversity in the biomass-amended treatments (Table 2), as already observed in our previous experiment (Silva et al., 2018) and other studies (Van Horn et al., 2011). The reduction of diversity and the replacement of indigenous species by rapidly growing “weeds” is a wide-spread consequence of eutrophication in plant and algal assemblages (Hillebrand and Sommer, 2000; Anderson et al., 2002; Hautier et al., 2009). Our data show that a comparable pattern is also found in microbial communities on GDM biofilms.

### More Synchronized Biofilm Communities Developing From River Water

Network analysis identified a clear separation between the experimental sites (Figure 5 and Supplementary Table S2). A significantly higher number of co-occurring OTUs (i.e., higher degree number; symbol size in Figure 5C) was found in those modules of the network that could be associated with ExpS from the position of the site-exclusive OTUs (Figure 5A,C and Supplementary Table S3). Thus, bacterial taxa in stream water biofilms in general had more synchronized growth patterns. This might be related to differences in the respective recruitment of biofilm-forming bacteria from lake and stream water. A considerable fraction of the planktonic bacteria in shallow streams such as Chriesbach are released into the water from the biofilms that form in the stream bed and are, in turn, seeds for new biofilm assemblages (Besemer, 2015). By contrast, potentially biofilm-forming bacteria in lake water may originate from a number of different habitats, such as the sediment surface (Dai et al., 2016), the periphyton (Crump and Koch, 2008; Hempel et al., 2008), terrestrial influx (Monard et al., 2016), suspended organic aggregates (Bižić-Ionescu et al., 2015), or aquatic animals (Grossart et al., 2010; Eckert and Pernthaler, 2014).

### MC Removing Bacteria in GDM Biofilms: Interplay of Seeding Effects and Biotic Interactions?

Habitat-specific “seeding effects” as outlined above might have contributed to the observed differences in MC degradation between ExpL and ExpS, reflecting the likely greater genotypic and functional diversity of MC degraders in lake water (Krishnan et al., 2018; Thees et al., 2018; Yang et al., 2018). The large *Planktothrix* population in Lake Zurich (Posch et al., 2012) provides an ample indigenous source of the toxin, and the degradation of toxins from cyanobacterial cells may occur in a variety of niches such as the water column or on sediment surfaces (Chen et al., 2008, Chen X. et al., 2010). One strategy to overcome such differences between feed water communities might thus be to initially seed GDM systems with immobilized bacteria expressing the MlrA enzyme, as has been proposed by Dziga et al. (2014).

Moreover, it is also conceivable that the more variably composed biofilm communities in ExpL allowed for the better establishment of facultative MC degraders than the more tightly knit “deterministic” communities of ExpS (Figure 5): only a single genotype could be unambiguously identified as ExpS-specific MC responder (i.e., >100 times more reads in ExpS than in ExpL, and >100 times more reads in ExpS WT than in ExpS MUT). It was the third most abundant OTU in our experiments (Table 1) and 98% identical to the obligate aerobe *Deinococcus cellulosilyticus* (Weon et al., 2007). This OTU only had one single partner with the same co-occurrence pattern [identical to a known sulfamethoxazole-degrading *Pseudomonas* sp. (Herzog et al., 2016)]. Presently, we can only speculate if the “MC responder” OTU was indeed also responsible for MC degradation. However, its conspicuous isolation within the otherwise highly synchronized stream water biofilm community might be an indication that more pronounced competitive interactions in ExpS impeded the establishment of a large variety of facultative MC degrading bacteria.

## Author Contributions

MS designed study, performed the experiments, and contributed to data evaluation and co-writing of the manuscript. PD assisted in experiments and discussion of data. ND assisted in experiments and contributed to discussion of data and to manuscript. EM contributed to discussion of data and to manuscript. JP designed study and contributed to data evaluation, discussion of data, and co-writing of the manuscript.

## Conflict of Interest Statement

The authors declare that the research was conducted in the absence of any commercial or financial relationships that could be construed as a potential conflict of interest.
