# Supplementary material for: Source Community and Assembly Processes Affect the Efficiency of Microbial Microcystin Degradation on Drinking Water Filtration Membranes
Source: Front Microbiol. 2019 Apr 18;10:843. doi: 10.3389/fmicb.2019.00843 (PMC6482319; doi:10.3389/fmicb.2019.00843)
Supplement: Supplementary file 1 [file Data_Sheet_1.PDF]

Supplement Material

Table S1 Bacterial community composition in the experimental systems (ExpL and ExpS) at the phylum level.

Phylum	Proportion of total reads [%] (proportion of all OTUs)				
	Total	ExpL	ExpS	Biomass-amended treatments	CTRL treatments
Proteobacteria	62 (52)	33 (30)	29 (39)	44 (35)	19 (37)
Bacteriodetes	11 (14)	7 (9)	4 (10)	8 (10)	3 (10)
Actinobacteria	7 (6)	3 (3)	4 (4)	1 (3)	6 (5)
Firmicutes	2 (4)	2 (2)	1 (3)	2 (2)	1 (3)
Parcubacteria	1 (0.2)	1 (0.2)	< 0.01 (0.1)	0.01 (0.1)	1 (0.2)
Acidobacteria	2 (2)	1 (1)	1 (1)	1 (1)	0.3 (14)
Chloroflexi	3 (4)	1 (2)	2 (3)	2 (2)	1 (2)
Spirochaetae	1 (1)	1 (1)	0.3 (1)	1 (1)	0.1 (1)
Deinococcus	6 (1)	1 (0.4)	5 (1)	6 (1)	0.1 (1)
Fibrobacteres	2 (0.4)	0.5 (0.2)	1 (0.4)	1 (0.3)	0.1 (0.3)
Chlamydiae	2 (7)	0.3 (3)	2 (5)	0.5 (4)	1 (5)
Chlorobi	1 (1)	0.2 (1)	0.4 (1)	0.3 (1)	0.3 (1)
Others	1 (8)	0.5 (4)	1 (6)	1 (5)	1 (6)

Network description:

Hundred-forty-nine OTUs affiliated with *Alphaproteobacteria* were present in 3 or more samples and 34 OTUs from this lineage were present in 9 or more samples. More than one third (13) of these site-independent OTUs were associated with module 2 (ExpS-Both). Six alphaproteobacterial OTUs in module 2 were associated with the *Caulobacterales*. The largest alphaproteobacterial OTU in this module was the *Rhodobacter sp.* 1 (AB515715 100 %)*;* it was the third largest OTU of the network and was negatively correlated with the second largest OTU, a gammaproteobacterium (*Pseudoxanthomonas sp*., JF166723, 100 %) from module 4 (ExpL-Biomass). Altogether, 78 gammaproteobacterial OTUs were present in at least 3 samples and approximately one third of them in 9 samples or more. The two largest OTUs of the network were gammaproteobacteria (*Lysobacter sp.*- KM083544, 100 %, and *Pseudoxanthomonas sp.*- JF166723, 100 %), both of them located in module 4 (ExpL-Biomass). Betaproteobacteria were overrepresented in the modules defined by biomass-exclusive OTUs (3 and 4), and the fifth largest OTU of the network (*Ideonella sp.*- AB769197,100 %) was situated in module 3. Sixty-seven betaproteobacterial OTUs were present in 3 or more samples and almost half of these occurred in 9 or more samples. Two betaproteobacteria (*Methyloversatilis universalis*, KJ147082, 100 %; and uncultured methlylotroph, JN869127, 100 %) had particularly high betweenness centrality; they represented a link between two 'lake' modules (4 and 5). Only 26 OTUs belonging to the deltaproteobacteria were present in at least 9 samples, but almost 5 times as many were present in ≥3 samples. Two deltaproteobacterial OTUs (*Phaselicystis sp.*- HQ609664, 100% and *Desulfobacterium sp.*- JN440844, 97%) with high betweenness centrality formed a link between modules 2 and 3 (ExpS-Both and ExpS-Biomass). All deltaproteobacteria in module 1 (mainly *Myxococcales*) had very high degree numbers. In general, the 'stream' modules (1, 2 and 3) harbored significantly more *Myxococcales* (78 % vs. 23 %) than the 'lake' modules 4 and 5 (Student's t-test, n=40, p <0.001) and *Bradymonadales* was only present in the 'stream' modules. By contrast, *Bdellovibrionales* (38 % vs. 34 %) formed a higher proportion of all deltaproteobacterial nodes in the 'lake' modules. The *Actinobacteria* were significantly more (58 % vs. 30 %) in those two modules (1 and 5) that were defined by their relationship with the CTRL treatments (Student's t-test, n= 50, p< 0.005).

Twelve OTUs were 'universal responders' to the experimental conditions in that they were present in all 18 samples (Table S2). Another 96 OTUs were found in 12 or more samples

and 198 in 9 or more samples. Only 2 OTUs (<1% of reads) were site-independent 'reliable biomass responders', i.e., exclusively present in all biomass-amended treatments (Table S2). Seven OTUs were treatment-independent 'reliable habitat indicators' for ExpS and 6 for ExpL, (i.e., exclusively present in all samples of a specific site, Table S2). These OTUs together formed 2.4% of all reads. Almost one tenth of all nodes in the network were 'reliable habitat and treatment indicators', in that they exclusively occurred in all 3 replicates of the CTRL treatment of ExpS (53 OTUs) or ExpL (14 OTUs). No 'reliable habitat and treatment indicators' were found for the biomass-amended treatments. Altogether, 49% and 65% of all nodes in the network were not site- or treatment-specific, respectively.

Table S2: OTUs in the co-occurrence network analysis that were universally present or limited to particular sites or treatments. Accession numbers of the closest database hits and % identity to these hits are reported in brackets

**Universally present OTUs**	**Site-independent 'reliable biomass responders'**	**Treatment-independent 'reliable habitat indicators' ExpS**	**Treatment-independent 'reliable habitat indicators' ExpL**
Lysobacter sp. (KM083544, 100 %)	*Stenotrophomonas rhizophila* (FJ529915, 100 %)	*Phaselicystis* sp. (HQ609664, 100%)	*Paucibacter* sp. (KM187599 , 100 %)
Acinetobacter sp. (LT223613, 100 %)	*Pseudohongiella* sp. (KC331307, 100 %)	*Desulfomicrobium* sp. (JFJP01041415, 100 %)	Chitinophagaceae (FJ546396, 100%)
Pseudoxanthomonas sp. (JF166723, 100 %)		*Stella humosa* (AJ535710, 100 %)	*Devosia* sp. (KM016328, 100 %)
Rhodobacter sp. 1 (AB515715, 100 %)		Sphingobacteriales (HE804134438, 100%)	Sphingobacteriales (FR667361, 100 %)
Cytophagaceae (JF703528, 100 %)		Parachlamydiaceae (FJ532292, 99.6 %)	*Legionella* sp. (DQ336999, 100 %)
Haliscomenobacter hydrossis (AJ784892, 100 %)		*Legionella* sp. (JF183838, 100 %)	uncultured methlylotroph (JN869127, 100 %)
Ideonella sp. (AB769197, 100 %)		*Haliangium* sp. (KF697567, 100 %)	
Methyloversatilis universalis (KJ147082, 100 %)			
Hydrogenophaga sp. (KT029151, 100 %)			
Comomonadacea (HM308520, 100 %)			
Reyranella sp. (FQ659574, 100 %)			
Rhodobacter sp. 2 (FJ612179, 100 %)			

Table S3 Distribution of OTUs that were exclusive for site and/or treatments across network modules (modularity class according to Blondel *et al.*, 2008).

Module	ExpS exclusive	ExpL exclusive	CTRL exclusive	Biomass-amended exclusive	Site independent	Treatment independent	Total	Module density (undirected)
1 CTRL ExpS	109	0	70	0	26	65	135	0.661
2 Both ExpS	47	0	2	0	77	122	124	0.139
3 Biomass ExpS	83	0	0	109	109	83	192	0.127
4 Biomass ExpL	0	56	0	54	83	85	139	0.06
5 both ExpL	0	52	15	0	35	72	87	0.167

Table S4 Top 10 most abundant OTUs in the enrichment experiment.

**Relative abundance** (%)	**Taxonomy** (Accession number and identity)	**Ratio anaerobic:aerobic**	**Ratio lake:stream**
17.4	*Limnobacter* (KJ549100,100 %)	1.6	1.05
10.0	*Acinetobacter* (LT223613, 100%)	0.025	2.49
9.4	*Brevundimonas vesicularis* (FM955876, 100 %)	0.014	0.48
8.5	*Thiobacillus* sp. (HQ640517, 100 %)	19316	149.91
6.5	*Paucibacter toxinovorans* (HG530247, 100 %)	0.018	0.09
5.3	*Pseudomonas oryzihabitans* (FJ824120, 100 %)	12172	2.21
4.4	*Methyloversatilis universalis* (KJ147082, 100 %)	0.001	0.08
4.4	*Methylotenera* sp. *N17* (JUGE01000001, 100 %)	Exclusively present in anaerobic enrichments	0.18
2.9	*Polynucleobacter* sp. (EU800806, 100 %)	6662	1.11
2.8	*Hydrogenophaga* sp. (KT029151, 100 %)	275.95	0.34

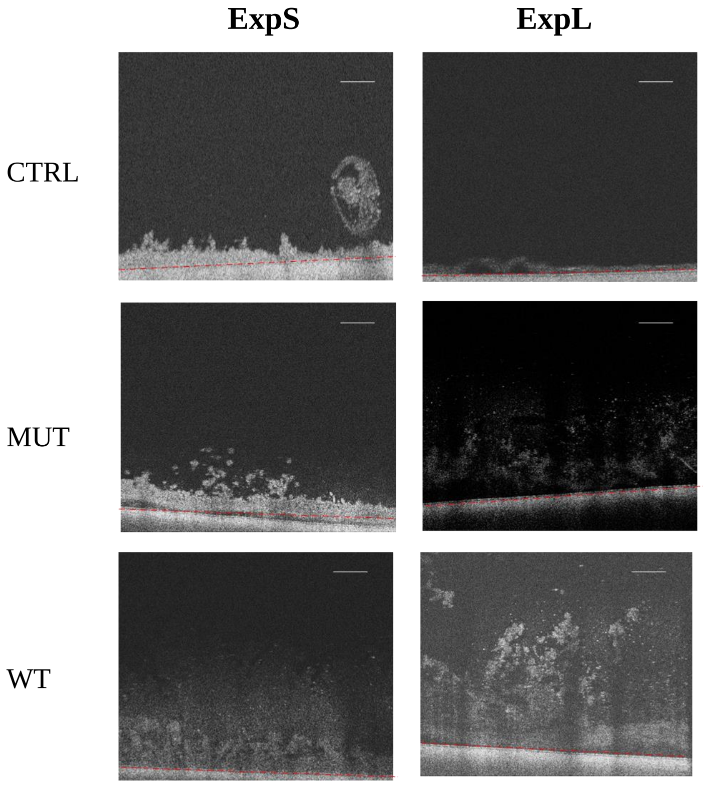

Figure S1 Optical coherence tomography (OCT) of membrane biofilms formed during ExpL and ExpS under constant transmembrane pressure. The structure was captured at the end of the experiment. The red line indicates the membrane surface.
